# Reviewed article: Rozman LM, Sartori AMC, Souza AA, Soarez PC. Discrepancy between official records of the Brazilian National Immunization Program Information System and the household survey about vaccination coverage in Cubatão, São Paulo: cross-sectional study, 2023. Epidemiol Serv Saude. 2025:34;e20240523

**DOI:** 10.1590/S2237-96222025v34e20240523.a

**Published:** 2025-08-11

**Authors:** Adelino Lima

**Affiliations:** Faculdade Estacio Alagoinhas

**Table:** 

Rating	Excellent	Good	Average	Below average	Poor
Interest	✓				
Quality	✓				
Originality		✓			
Overall		✓			

**Table:** 

Questionnaire	Yes	No	Not applicable
Does the manuscript contain new and significant information to justify publication?	✓		
Does the Abstract (Summary) clearly and accurately describe the content of the article?	✓		
Is the problem significant and concisely stated?	✓		
Are the methods described comprehensively?	✓		
Are the interpretations and conclusions justified by the results?	✓		
Is adequate reference made to other work in the field?	✓		

## Manuscript Structure

Length of article is: Adequate

Number of tables is: Adequate

Number of figures is: Adequate

Please state any conflict(s) of interest that you have in relation to the review of this paper (state “none” if this is not applicable): None

## Comments

O artigo aborda um tema atual e relevante, considerando os desafios enfrentados pelo sistema de saúde brasileiro. O foco na confiabilidade dos dados vacinais possui grande relevância. As recomendações aqui apresentadas têm apenas o propósito de contribuir para possíveis melhorias do escrito: Em relação aos resultados, estes são apresentados de maneira clara, com tabelas que complementam a descrição textual. No entanto, uma explicação mais detalhada sobre como interpretar os valores de Kappa e ICC seria útil. A discussão apresenta bem os desafios na coleta de dados e na implementação do sistema e-SUS. Sugiro ampliar a comparação com outros estudos internacionais, fortalecendo a generalização dos achados. Pontos Fortes: A amostra é representativa e a metodologia robusta. A discussão sobre a interoperabilidade e a integração de sistemas de saúde é o ponto alto do artigo. Limitações: Poderia explorar mais as implicações práticas dos resultados para a gestão municipal e federal. Não houve discussão profunda sobre possíveis soluções para a baixa qualidade de dados em municípios com menor infraestrutura. Reorganizar parágrafos longos para aumentar a fluidez da leitura. Explorar mais detalhadamente as possíveis ações práticas para corrigir os problemas encontrados.

